# The Function and the Affecting Factors of the Zebrafish Gut Microbiota

**DOI:** 10.3389/fmicb.2022.903471

**Published:** 2022-06-02

**Authors:** Pingping Li, Jinhua Zhang, Xiaoyi Liu, Lu Gan, Yi Xie, Hong Zhang, Jing Si

**Affiliations:** ^1^Institute of Modern Physics, Chinese Academy of Sciences, Lanzhou, China; ^2^Key Laboratory of Heavy Ion Radiation Biology and Medicine of Chinese Academy of Sciences, Lanzhou, China; ^3^College of Life Sciences, University of Chinese Academy of Sciences, Beijing, China; ^4^College of Life Science, Lanzhou University, Lanzhou, China; ^5^Advanced Energy Science and Technology Guangdong Laboratory, Huizhou, China

**Keywords:** gut microbiota, zebrafish, functions, factors, host health

## Abstract

Gut microbiota has become a topical issue in unraveling the research mechanisms underlying disease onset and progression. As an important and potential “organ,” gut microbiota plays an important role in regulating intestinal epithelial cell differentiation, proliferation, metabolic function and immune response, angiogenesis and host growth. More recently, zebrafish models have been used to study the interactions between gut microbiota and hosts. It has several advantages, such as short reproductive cycle, low rearing cost, transparent larvae, high genomic similarity to humans, and easy construction of germ-free (GF) and transgenic zebrafish. In our review, we reviewed a large amount of data focusing on the close relationship between gut microbiota and host health. Moreover, we outlined the functions of gut microbiota in regulating intestinal epithelial cell differentiation, intestinal epithelial cell proliferation, metabolic function, and immune response. More, we summarized major factors that can influence the composition, abundance, and diversity of gut microbiota, which will help us to understand the significance of gut microbiota in regulating host biological functions and provide options for maintaining the balance of host health.

## Introduction

Gut microbiota is the collective community of microbiota that reside in the gastrointestinal tract of the host (Lozupone et al., 2012). Gut microbiota has been described as an important functional host “organ” (Stephens et al., 2016; Lankelma et al., 2017). There is growing evidence that gut microbiota is closely associated with host health (Mangiola et al., 2016; Patterson et al., 2016; Angelucci et al., 2019; Gomaa, 2020) and plays a role in several aspects including intestinal epithelial cell differentiation (Darby et al., 2020), intestinal epithelial cell proliferation (Darby et al., 2020), nutrient metabolism (Lopez et al., 2020) and immune response (Stappenbeck et al., 2002; Yang et al., 2017; Murdoch and Rawls, 2019; Lopez et al., 2020), xenobiotic metabolism (Darby et al., 2020), angiogenesis (Stappenbeck et al., 2002; Franks, 2013), and host growth (Schwarzer, 2018; Schwarzer et al., 2018; Consuegra et al., 2020).

Zebrafish is an excellent model for studying gut microbiology. First, the advantages of high fecundity, small size, short reproductive cycle, rapid growth, and easily achievable rearing conditions make it possible to provide many zebrafish samples for experimental studies (Garcia et al., 2016; Stagaman et al., 2017). Second, zebrafish share a high degree of physiological and genetic homology with humans, with approximately 2,601 (82%) human disease-associated genes and 18,029 (71.4%) human genes having at least one zebrafish homolog (Howe et al., 2013). Third, zebrafish larvae can be used for large-scale germ-free (GF) studies (Rawls et al., 2004; Hill et al., 2016). The transparency of zebrafish larvae facilitates comprehensive sampling and real-time imaging of gut microbiota (Stephens et al., 2015), which makes it easier to study host–microbiota interactions and microbiota assemblages (Rawls et al., 2004; Wong et al., 2015). Fourth, the use of fluorescent protein expressing bacteria and/or fluorescent protein expressing zebrafish (Rawls et al., 2004), transgenic zebrafish models, and mutant zebrafish strains, microbiota transfer techniques (Guo et al., 2019), the construction of knockout zebrafish (Kok et al., 2015), and the use of genus-specific probes for microbial distribution have broadened the applicability of zebrafish in gut microbiota studies (Bates et al., 2006). These advantages have made zebrafish a common research model used by scientists for the study of gut microbiota. These growing results of zebrafish in gut microbiota can help us understand the microbiota–host interactions better (Milligan-Myhre et al., 2011; Semova et al., 2012; Liu et al., 2016).

In our review, we focus on the relationship between gut microbiota and host health, so we summarize the role of gut microbiota in intestinal epithelial differentiation, enterocyte proliferation, host nutrient metabolism, and immune response. We also list the major factors that influence gut microbiota composition, abundance, and diversity. This can help us provide insight into the importance of the involvement of gut microbiota in the regulation of the host organism and provide some useful therapeutic options for maintaining gut ecological homeostasis and host health.

## Physiological Functions of Gut Microbiota in Zebrafish

### Intestinal Epithelium Differentiation

Gut microbiota influences intestinal epithelial cell differentiation through lipopolysaccharide (LPS; Bates et al., 2006). LPS is a component of the outer membrane from Gram-negative bacteria. During the establishment of microbiota, LPS can promote a dramatic increase in intestinal alkaline phosphatase (IAP, a classical marker of intestinal epithelial cell maturation; Beutler and Rietschel, 2003; Bates et al., 2006; Swain et al., 2008). In GF zebrafish larvae without gut microbiota, it exhibits immature brush border enzyme activity in the intestine (Bates et al., 2006). At the same time, the gut of GF zebrafish larvae is blocked in specific aspects of differentiation and its function is altered in specific aspects (Bates et al., 2006). In contrast, the opposite result was observed in conventionally reared (CV) zebrafish larvae due to the presence of gut microbiota in their gut (Bates et al., 2006). Therefore, the ability of gut microbiota to promote intestinal epithelial cell differentiation is mainly due to the induction of alkaline phosphatase activity in the zebrafish gut by gut microbiota through bacterial LPS. In addition, exposure to exogenous LPS was also sufficient to increase IAP transcript levels in GF zebrafish larvae (Bates et al., 2007). Also, there is evidence that in the absence of microbiota, the alkaline phosphatase activity in GF zebrafish is significantly lower than in the gut of CV zebrafish and reverses the GF phenotype by introducing bacteria later in development (Bates et al., 2006).

Gut microbiota can regulate the differentiation of intestinal epithelial cells through indirect effects. The farnesoid X receptor (*fxr*) is a nuclear receptor and transcription factor with regulatory physiological functions, including the regulation of intestinal epithelial cell differentiation (Lefebvre et al., 2009; Vitek and Haluzik, 2016; Chiang et al., 2017; Wen et al., 2021). In zebrafish, gut microbiota alters primary bile salts to regulate *fxr*-mediated signaling and thus regulate the differentiation of anterior absorptive enterocytes (Wen et al., 2021). In conclusion, it is clear that gut microbiota plays an important role in the differentiation of zebrafish intestinal epithelial cells.

### Epithelial Cell Proliferation

Germ-free zebrafish lacking gut microbiota have reduced proliferation ability of epithelial cells. The minichromosome maintenance genes (*mcm*), as a proliferative and prognostic molecular marker, its expression varies with the difference of gut microbiota in CV zebrafish. A recent study provide the evidence that gut microbiota stimulates zebrafish intestinal epithelial cell proliferation by increasing the expression of genes involved in DNA replication and cell division, such as proliferating cell nuclear antigen (*pcna*, *a* biomarker of epithelial cell renewal; Rawls et al., 2006), thymidylate kinase (*Dtymk*), origin-recognition complex subunit 4 (*Orc4l*), and ribonucleotide reductase subunit M2 (*Rrm2*), *mcm2*, *mcm3*, *mcm5*, and *mcm6* (Rawls et al., 2004).

### Nutrient Metabolism

It has been reported in mice that gut microbiota regulates intestinal absorption and metabolism (Backhed et al., 2004). A recent study found a similar effect in zebrafish gut microbiota (Iqbal and Hussain, 2009). Microbial-deficient zebrafish is impaired in their ability to use nutrients. Diet-induced changes in microbiota composition may affect fat absorption (Semova et al., 2012). Dietary fat in the form of triglycerides is first digested by lipase in the intestinal lumen, releasing free fatty acids (FFAs) and mono-triglycerides. Immediately afterwards, the free fatty acids and mono-triglycerides are absorbed by the intestinal epithelial cells (Iqbal and Hussain, 2009). Later, the fatty acids are esterified to triglycerides and temporarily stored as cytoplasmic lipid droplets (LDs). Finally, some LDs may be incorporated into celiac particles for secretion into the lymphatic fluid, while others may be released into the circulation (Iqbal and Hussain, 2009). A published study has shown that gut microbiota increases the transport of fatty acids to extra-intestinal tissues (e.g., liver), which can promote the accumulation of LDs in the intestinal epithelium and ultimately the absorption of fatty acids (Semova et al., 2012). In microbiota-deficient zebrafish, the expression of genes involved in lipid metabolism is significantly influenced by gut microbiota (Falcinelli et al., 2015).

Meanwhile, gut microbiota regulates cholesterol metabolism and trafficking in zebrafish. A study showed that gut microbiota in the zebrafish digestive tract can upregulate apolipoprotein B (*Apob*), which catalyzes the first step in cholesterol catabolism and bile acid biosynthesis. Upregulation of *Apob* has implications for cholesterol catabolism and bile acid biosynthesis (Rawls et al., 2004). Gut microbiota regulates host lipid processing by decreasing transcript expression levels of genes involved in cholesterol and triglyceride metabolism (*fit2*, *agpat4*, *dgat2*, *mgll*, *hnf4α*, s*cap*, and *cck*; Falcinelli et al., 2015). Another evidence of the effect of gut microbiota on nutritional metabolism in zebrafish is that the GF zebrafish gut has a functional defect in protein uptake (Rawls et al., 2006). Microbe-deficient zebrafish upregulates the expression of solute carrier family genes (e.g., *SLC7a3*, *SLC38a4*, and *SLC15a2*), thereby increasing the transport of amino acids or peptides that are used to compensate for the lack of protein intake (Rawls et al., 2004).

Therefore, it can be concluded that gut microbiota has an important role in regulating host nutrient absorption, including its role in regulating host fatty acid absorption, cholesterol metabolism, and protein absorption.

### Immune Responses

Gut microbiota may be a factor in triggering the host immune response, that is, gut microbiota modulates immune responses (Rawls et al., 2004; Yang et al., 2017). LPS, a component of the outer wall layer of gram-negative bacteria (Swain et al., 2008), increases the transcription of inflammatory factors in zebrafish and causes characteristic tissue lesions, such as infectious shock, and even death (Mottaz et al., 2017). Toll like receptors (TLRs) is a pattern recognition receptor for innate immunity. All orthologs of mammalian TLR genes can be found in zebrafish, and both mammalian and zebrafish TLRs have the same characteristic structure (Jault et al., 2004). LPS exerts the toxic effects *via* overstimulating TLRs innate immune signaling (Jault et al., 2004; Swain et al., 2008), thus leading to pathogenic inflammatory response (Rawls et al., 2006; Bates et al., 2007).

**Figure 1:**
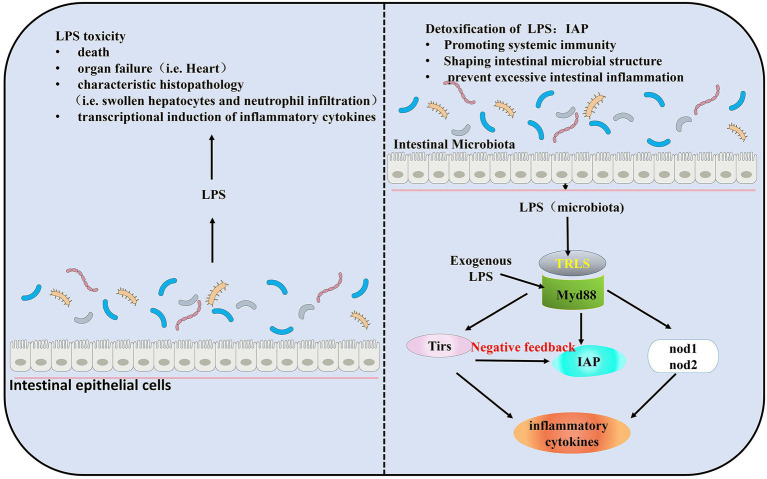
Intestinal homeostasis during the establishment of gut microbiota colonization by Myd88, toll like receptors (TLRs). Lipopolysaccharide (LPS) is required for the induction of intestinal alkaline phosphatase (IAP) activity and triggers intestinal inflammation through TLRs and Tnf signaling. IAP transcript levels were significantly elevated in the presence of gut microbiota. Exposure to exogenous LPS increases IAP transcript levels in germ-free (GF) larval zebrafish and results in higher than normal IAP levels in conventionally reared (CV) larval zebrafish. In a negative feedback loop formed by TLRs, IAP, and LPS, IAP dephosphorylates LPS, and TLRs and Tnf are reduced by LPS dephosphorylation, thereby preventing excessive intestinal inflammation.

Gut microbiota induces the expression of the IAP located at the intestinal brush border to detoxify the potential toxicity of LPS. LPS is necessary for the induction of IAP activity (Bates et al., 2006). LPS mediates AP activity in the intestine, while IAP dephosphorylates LPS (Bates et al., 2007; Fabbiano et al., 2018). IAP can act as an antidote to decrease the toxicity of the LPS. The response to dephosphorylated LPS is induced during microbiota establishment and plays a key role in promoting intestinal mucosal tolerance in zebrafish and in promoting mucosal tolerance to intestinal bacteria (Bates et al., 2006). IAP is considered valuable for its ability to prevent excessive immune responses through negative feedback regulation of TLRs. TIR-domain containing adapters MyD88, TLRs, IAP, and the pro-inflammatory cytokines (such as tumor necrosis factor TNF) comprise a signaling pathway to maintain the homeostatic balance of the intestinal (Jault et al., 2004; Bates et al., 2007; Brugman et al., 2009; Figure 1).

**Figure 2:**
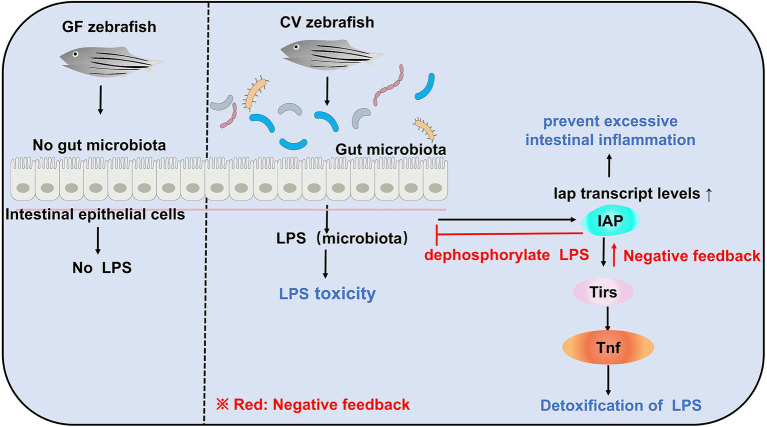
Gut microbiota can induce LPS toxicity in CV zebrafish. In GF zebrafish, the gut is sterile and therefore does not induce LPS toxicity. Although gut microbiota in CV zebrafish can induce LPS toxicity, e.g., infectious shock, and even death, there is also negative feedback regulation in CV zebrafish to maintain host homeostasis.

Due to the lack of gut microbiota in zebrafish larvae, there is no LPS production *in vivo* to induce IAP activity, which further inhibits the transcription of inflammatory factors and ultimately suppresses the formation of neutrophils in the intestinal tract of GF zebrafish. In contrast, in GF zebrafish larvae, the addition of exogenous LPS can induce IAP activity, thereby avoiding intestinal inflammation (Figure 2; Bates et al., 2007). Another evidence that demonstrates microbial regulation of the immune response is that colonization of microbiota in zebrafish increases the expression of a biomarkers of the innate immune response saa (serum amyloid a; Rawls et al., 2006). From the perspective of this establishment of intestinal homeostasis, a strategy to slow down enteritis can be developed: inhibition of innate immune responses, such as those mediated by TIR-domain containing adapters Myd88 (Jault et al., 2004; Bates et al., 2007), leads to alteration of the intestinal microbial community and ultimately to a good effect of reducing intestinal inflammation (Chu and Mazmanian, 2013).

## Factors Affecting Gut Microbiota

**Figure 3:**
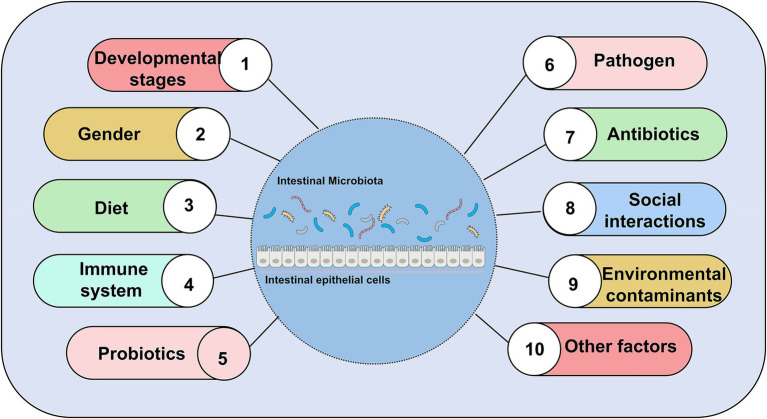
Major factors affecting gut microbial composition, abundance, and diversity, excluding gender.

Zebrafish has a complex commensal bacteria community in its digestive tract (Zang et al., 2019). At the very least, 500–1,000 different species of bacteria colonize the gut of zebrafish (Zhang Q. et al., 2019). Bacterial colonization of the zebrafish gut after hatching coincides with the differentiation of the digestive tract (Bates et al., 2006). There are temporal differences of intestinal microbial distribution (Bates et al., 2006; Rawls et al., 2006; Falcinelli et al., 2015; Hill et al., 2016; Yang et al., 2017; Catron et al., 2019). In terms of phylum, the microbial community of zebrafish is usually dominated by *Proteobacteria*, *Firmicutes*, and *Fusobacteria* (Liu et al., 2016; Zhou et al., 2018). With the maturation of the microbiota, the microbial communities become more complex and diverse. Some differences in the composition of the gut microbial community of zebrafish may vary with host factors and exogenous factors. A number of host factors contain development stage (age), gender, and host’s immune system. Exogenous factors that include diet composition, feeding practices (temperature, feeding schedule, salinity), pathogens (infectious microbiota and viruses), antibiotic use, housing infrastructure, and water chemistry can affect the composition of zebrafish gut microbiota (Brugman et al., 2009; Roeselers et al., 2011; Mottaz et al., 2017; Yang et al., 2017). Noteworthy, gender had no significant effect on gut microbiota in zebrafish (Roeselers et al., 2011; Liu et al., 2016). Male and female zebrafish gut microbiota had similar bacterial composition. Sexual differentiation occurs in zebrafish during 35–75 days post-fertilization (dpf). During sexual differentiation in zebrafish, the diversity of gut microbiota changes despite keeping the diet and environment unchanged. However, the homogeneity of the gut community remains relatively unchanged during host development (Stephens et al., 2016). In our review, we reviewed nearly 20 years of published articles to summarize several factors that influence composition, abundance, diversity of gut microbiota in zebrafish (Figure 3).

### Different Developmental Stages

**Figure 4:**
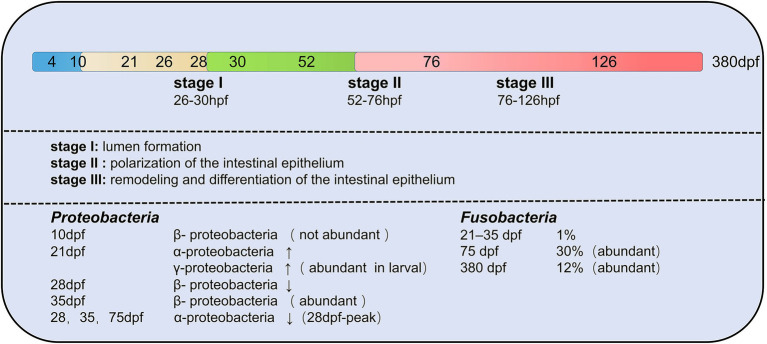
Changes in the composition of gut microbiota of zebrafish at different developmental stages. A total of three transitions of gut microbiota occurred throughout the developmental stages. The first transition of gut microbiota occurs at 10 days post-fertilization (dpf), the second transition of gut microbiota from embryo to juvenile occurs at 35–75 dpf in zebrafish, and the last transition of gut microbiota is from juvenile to early adult at 76 dpf–126 hpf. *Proteobacteria* and *Fusobacteria* are the two main core groups of zebrafish at different developmental stages, but both show different changes (increasing or decreasing) in different developmental stages.

The composition of zebrafish gut microbiota is different at different developmental stages and inter-individual differences increase with developmental period (age; Stephens et al., 2016). The developmental period of zebrafish is categorized into larval, juveniles and adults. In all stages of the zebrafish life cycle, gut microbiota of zebrafish is dominated by members of the phylum *Proteobacteria* (Bates et al., 2006; Rawls et al., 2006; Falcinelli et al., 2015; Yang et al., 2017; Catron et al., 2019; Lopez et al., 2020). *γ-proteobacteria* and *α-proteobacteria* are both *Proteobacteria*, but *γ-proteobacteria* is abundant in the gut of juvenile fish and *α-proteobacteria* increase in development to 21 dpf (juveniles; Stephens et al., 2016). During the larval stage, gut microbiota differs minimally between individuals. During the day of mouth opening, bacteria are less abundant in the mouth, pharynx, esophagus, and proximal intestine by 4 dpf (Bates et al., 2006). Between 4 and 8 dpf, the abundance of gut microbiota increased. Individual differences gradually increase with age. *Aeromonas* and *Pseudomonas* species predominate in the zebrafish embryo and larvae (Bates et al., 2006). The phylum *Fusobacteria* is more prevalent in the adult stage of zebrafish (Roeselers et al., 2011). Therefore, it can know that the composition of the zebrafish gut microbiota rapidly differentiates during early host development (Wong et al., 2015; Figure 4).

### Diet

Gut microbiota members correlated with dietary composition have been found in humans and other mammals (Ley et al., 2008; Muegge et al., 2011). Recent studies have shown that the zebrafish gut microbiota changes in response to changes in diet (Semova et al., 2012; Koo et al., 2017; Yang et al., 2017). Dietary changes are mainly reflected in differences in feeding or not, diet type, and diet density (Semova et al., 2012; Koo et al., 2017; Osimani et al., 2019). Feeding promotes bacterial diversity in the gut of zebrafish (Semova et al., 2012). Compared to zebrafish fed a sterile diet or fed a low-calorie diet, gut microbial diversity and abundance were significantly lower in the gut of non-fed zebrafish (Semova et al., 2012). It was found that the addition of the bioactive compounds chlorogenic and caffeic acids to the diet of zebrafish has an effect on enterobacteriaceae bacteria (Osimani et al., 2019). Eating food contaminated with graphene family materials leads to gut microbiota dysregulation in zebrafish (Zheng et al., 2019). The fat density in the diet affects the composition of gut microbiota of zebrafish (Semova et al., 2012; Wong et al., 2015; Falcinelli et al., 2017). Feeding diets with different fat densities (dietary fat levels) have a significant effect on the diversity of gut microbiota (Falcinelli et al., 2017). During 35 and 70 dpf, there are significant differences in gut microbiota of zebrafish fed high- and low-fat diet (Wong et al., 2015). In conclusion, whether fed or not, the diet type and diet density affects the abundance, composition, and diversity of gut microbiota in zebrafish.

### Immune System

Immunity system interacts with gut microbiota by affecting the composition and diversity to affect host health. This conclusion has been confirmed in humans and mice (Dimitriu et al., 2013; Davenport et al., 2015; Zhang et al., 2015; Yoo et al., 2020). Recent studies have shown that the immune system is an influential factor affecting the composition and diversity of the zebrafish gut microbiota (Mottaz et al., 2017; Stagaman et al., 2017; Yang et al., 2017). Zebrafish begin to possess an adaptive immune response 5 dpf, and at this time lymphocytes in the gut begin to function in the adaptive immune process (Coronado et al., 2019). Adaptive immunity inhibits the growth of *Vibrio* in the intestine. Compared to wild-type zebrafish with adaptive immune, *Rag1*-deficient zebrafish lacking adaptive immunity have a different gut microbial composition and a large number of *Vibrio* species thriving in their intestines (Brugman et al., 2014). T lymphocytes play an important role in adaptive immunity (Hohn and Petrie-Hanson, 2012; Tian et al., 2017). When T lymphocytes are transferred to *Rag1*-deficient zebrafish, the growth of *Vibrio* species in the gut of Rag1-deficient zebrafish is suppressed (Brugman et al., 2014). This is strong evidence that immunity affects the gut microbial composition of the gut, especially adaptive immunity.

### Probiotics

Probiotics alter the composition and relative abundance of gut microbiota (Gioacchini et al., 2012; Falcinelli et al., 2015; Zhang Z. et al., 2019; Chen et al., 2020). The addition of probiotics alters the abundance of the host’s original gut microbiota. *Lactobacillus rhamnosus* treatment significantly reduced the lower gut microbial diversity, higher relative abundance of *Firmicutes* and *Lactobacillus*, and lower relative abundance of *Mycobacterium* (fish pathogen; Falcinelli et al., 2015). The addition of exopolysaccharides from the probiotic *Lactobacillus casei* BL23 increased the abundance of *proteobacteria*, while the abundance of *Fusobacteria* decreased (Zhang Z. et al., 2019). The addition of *Lactobacillus plantarum* ST-III not only restored the species and quantity of intestinal microbiota but also inhibited the growth of saprophytic bacteria (Zang et al., 2019). The probiotic *L. rhamnosus* IMC 501 can alleviate high-fat diet-induced intestinal microbial disorders (Falcinelli et al., 2017). And Table 1 lists other beneficial bacteria that also regulate the homeostasis of the gut.

**Table 1:** Various probiotic with the change intestinal microbial composition.

Species/strains	Zebrafish	Effects/outcomes	References
*Aeromonas hydrophila* NJ-1	GF	Protected zebrafish from pathogenic infection	Guo et al., 2019
*Lactobacillus rhamnosus* GG	Zebrafish	Conferred higher protection against inflammation	Sireswar and Dey, 2019
*Lactobacillus plantarum* ST-III	Zebrafish	Recovered the species and number of microbiota in the intestines of zebrafish, and inhibited toxin production by saprophytic bacterial growth	Zang et al., 2019
*Lactobacillus rhamnosus*	Zebrafish	Altered gut microbiota community and highlighted the potential of probiotics to attenuate HFD-related metabolic disorder	Falcinelli et al., 2017
*Lactobacillus rhamnosus*	Larvae	Increased the abundance of *Firmicutes* sequence and reduced the abundance of *Actinobacteria*	Falcinelli et al., 2015
*Lactobacillus plantarum*	Adult	Altered the β-diversity of gut microbiota	Falcinelli et al., 2015
*Lactobacillus rhamnosus* IMC 501	Zebrafish	Significantly increased the abundance of *Firmicutes* and decreased *Proteobacteria*	Borrelli et al., 2016

### Social Interactions

Social interactions between hosts may promote microbial dispersal. This is demonstrated in a study in which splitting between hosts could greatly affect the diversity and composition of the zebrafish gut microbiota (Burns et al., 2017). The microbiota composition in the gut of zebrafish reared alone differed more than between co-housing zebrafish within a tank, implying that co-housing-induced social interactions results in more similar gut microbiota composition in co-housing hosts (Stagaman et al., 2017).

### Pathogen

The pathogen infection disrupts the balance of the pre-existing gut microbiota. *Pseudocapillaria tomentosa* infection disrupts the composition of zebrafish gut microbiota (Gaulke et al., 2019). Also, a published research study showed that pathogen challenge can affect the abundance of gut microbiota. Following an attack by *Aeromonas hydrophila* (aquatic pathogen), the bacterial abundance of zebrafish gut microbiota is altered, as evidenced by a decrease in beneficial bacteria and an increase in harmful bacteria in the gut (Yang et al., 2017).

### Antibiotics

The use of antibiotics affects bacterial diversity, composition, and abundance. Most antibiotics cause intestinal alterations, which usually include effects on the microbiota (depletion of the commensal microbiota), direct effects on host tissues, and effects on antibiotic-resistant microbiota, which have been reported in mice (Ubeda et al., 2010; Sorbara et al., 2019; Keith et al., 2020). In zebrafish, the application of antibiotics, such as colistin sulfate, penicillin, streptomycin, kanamycin, oxytetracycline, and vancomycin hydrochloride, had an effect on the gut microbial composition (Brugman et al., 2009; Willing et al., 2011; Zhou et al., 2018; Stressmann et al., 2021). A study also reported that environmental antibiotics can also impair intestinal health in zebrafish (Zhou et al., 2018). Among these, bacterial diversity or composition is more susceptible to some low concentrations of antibiotics (Zhou et al., 2018). After treatment with penicillin, streptomycin, and kanamycin administration, the homogeneity of the bacterial community of zebrafish was reduced. The number of antibiotic-sensitive bacteria may decrease with increasing antibiotic treatment, while the number of antibiotic-resistant bacteria increases (Stressmann et al., 2021). The addition of oxytetracycline exposure also disturbs gut microbiota of zebrafish, resulting in significant changes in the composition of gut microbiota (Zhou et al., 2018). Overall, it is clear from these studies that antibiotics are a factor that affects the composition, abundance, and diversity of gut microbiota.

### Environmental Contaminants

The host gut is a sensitive organ to environmental contaminants (Sun et al., 2019). Exposure to environmental pollutants tends to induce disorders of gut microbiota, intestinal inflammation, disorders of intestinal metabolism, and other adverse conditions, thus affecting the health of the host (Jin et al., 2017; Chen et al., 2018). Bactericides, microplastics or other common environmental contaminants can affect the composition, diversity or abundance of gut microbiota. 9-nitroanthracenes (microplastics) altered the dominant abundance of five major bacterial phyla (*Proteobacteria*, *Firmicutes*, *Fusobacteriota*, *Bacteroidota*, and *Verrucomicrobiota*; Zhang et al., 2021). Imazalil (IMZ) is a highly effective fungicide more commonly applied in aquatic systems. IMZ exposure resulted in a significant decrease in the abundance of Akkermansia, Alistipes, and Bacteroides in the gut of zebrafish (Jin et al., 2017). Previously, a study also found that IMZ also affected intestinal bacteria dysbiosis in mice (Jin et al., 2016). Carbendazim (fungicide) significantly changed gut microbiota composition (Bao et al., 2020). Chronic triclosan (bactericide) exposure affected the gut microbiota of zebrafish, mainly by influencing the abundance of gut microorganisms, where chronic triclosan exposure significantly increased the abundance of *Proteobacteria* (Zang et al., 2019). Microcystin-LR and glyphosate are common carcinogens present in aquatic ecosystems. Available studies have shown that microcystin-LR and glyphosate exposure significantly alters microbial communities in the zebrafish gut and induces alterations in the expression levels of apoptosis-related genes (Ding et al., 2021). Environmental chemical contaminant diethylhexyl phthalate and organochlorine dieldrin affects the diversity (Wang et al., 2022) and the abundance of intestinal microbiota (Hua et al., 2021), respectively. Polybrominated diphenyl ethers (PBDEs) is an environmental contaminant commonly found in furniture, textiles, appliances, and electronics. Acute exposure to PBDE-71 affects host gut health and significantly alters the composition of the zebrafish gut microbiota. This is because PBDE-71 exposure induces the host gut to establish a suitable gut community to adapt to the adverse effects of PBDE-71 exposure (Chen et al., 2018). From the above study data, it is known that the exposure to environmental pollutants is one of the factors affecting the composition, abundance, and diversity of gut microbiota.

Interestingly, gut microbiota can significantly modulate exogenous substance-induced toxicity by metabolically activating or inactivating exogenous substances (Jeong et al., 2013). Azoreductases, nitroreductases, β-glucuronidases, sulfatases, and β-lyases are involved in the metabolism of contaminants by the intestinal microbiota (Sousa et al., 2008; Claus et al., 2016). Available data suggest that biomarkers related to neurotransmission, epithelial integrity, inflammation, oxidative stress, and detoxification capacity correlate with the abundance of some genera. For example, streptococcus is positively correlated with intestinal ROS levels (oxidative stress), and conversely, Prevotella is negatively correlated with intestinal ROS (Chen et al., 2018). Members of cytochrome P450, *Cyp1a1*, *Cyp2b6*, and *Cyp2c19* are involved in xenobiotic metabolism and have increased expression in the digestive tract of GF zebrafish (Denison and Whitlock, 1995; Rawls et al., 2004). Additional studies also found that increased cytochrome P450, and GST contribute to the resolution of hygromycin toxicity. Notably, the GF zebrafish digestive tract may be less able to detoxify dietary components and other components of the environment (Rawls et al., 2004). The data above suggest that contaminants affect the intestinal bacterial flora. Conversely, the gut microbiota may modulate the toxicity of contaminants to the host.

### Other Factors

Endocrine disrupting chemicals affect the zebrafish gut microbiota (Bates et al., 2007). Estradiol (E2) and bisphenol A (BPA) are two endocrine-disrupting chemicals. Treatment of zebrafish with E2 and BPA ultimately led to dysbiosis of gut bacterial ecology in zebrafish, but the results showed that this was independent of the sex of the zebrafish and may be mainly related to changes in host lipid metabolism (Roeselers et al., 2011; Liu et al., 2016). Occasionally, gut microbiota of zebrafish varies depending on the aquaculture system (i.e., separated housing units; Stagaman et al., 2017; Catron et al., 2019), temperature, and the strain of zebrafish (Stephens et al., 2016). Microbial assembly of zebrafish is strongly influenced by water quality (Liu et al., 2016) and water samples (Stagaman et al., 2017). Both high and low environmental temperatures can affect the structure of zebrafish gut microbiota. Among them, high temperature has a more drastic effect on the structure of zebrafish gut microorganisms (Wang et al., 2022). A more detailed description of the factors affecting the composition and abundance of gut microbiota are be listed in Tables 2, 3.

**Table 2:** Factors affecting gene expression by affecting gut microbiota in zebrafish.

Factors	Zebrafish	Microbiota-related function	Gene expression	References
*Vibrio* sp.	CV	Innate immune response	Upregulated (*tlr4*, *tlr5*, *nod1*, *nod2*, *Myd88*, *tnfa*, *tnfβ*, *il1β*) Downregulated (tlr2, NF-κB pathway genes, JNK/AP-1 pathway genes)	Xin et al., 2020
*Vibrio* sp.	GF	Innate immune response	Upregulated (*Myd88*, *tlr2*, *tlr5*, *nod1*, *nod2*) Downregulated (NF-κB pathway genes, JNK/AP-1 pathway genes)	Xin et al., 2020
*Aeromonas* sp.	CV	Innate immune response	Upregulated (*nod1*, *nod2*, *tnfa*, *tnfβ*, *il1β*, *Myd88*, *nfκb2*, *jund*)	Xin et al., 2020
IMZ	Male adult	Glycolysis and lipid metabolism	Downregulated (*Aco*, *Cpt*1, *Acc1*, *Srebp1a*, *Fas*)	Jin et al., 2017
CAgNC	Zebrafish	Immune and mucin response	Upregulated (*TNF-α*, *IL-10*, *IL-12*, *IRF-1*, *Defbl1*, *Lyz*)	Udayangani et al., 2017
Eos	GF	Immune response	Upregulated (*il1β*, *Claudin1*, *Occludin2*)	Ran et al., 2016
*Lactobacillus rhamnosus*	Larvae	Cholesterol and triglycerides metabolism	Upregulated (*fit2*, *agpat4*, *dgat2*, *mgll*, *hnf4α*, *scap*, *cck*)	Falcinelli et al., 2015

*NF-κB pathway genes: *nfκb1*, *nfκb2*, *rela*, *relb*, and *c-rel*; JNK/AP-1 pathway genes: *c-fos*, *c-jun*, *jun*, *jund*, *junbb*, *junba*, and *june*; CagNC, chitosan silver nanocomposites; Eos, Essential oils; *tlr2*, *tlr4*, *tlr5*, and *nfκb2*: innate immunity-related gene; *nod1* and *nod2*: NLRs-related genes, and inflammatory cytokines; *Myd88*: innate immune signal transduction adaptor; NF-κB pathway genes, JNK/AP-1 pathway genes, innate immunity-related genes; *tnfa*, *tnfβ*, and *il1β*: TLRs-related genes; jund, JNK/AP-1 pathway genes; *Aco*, *Cpt1*, *Acc1*, *Srebp1a*, and *Fas*: glycolysis and lipid metabolism genes; *TNF-α*, *IL-10*, *IL-12*, *IRF-1*, *Defbl1*, and *Lyz*: immune and mucin genes; *il1β*, *Claudin1*, and *Occludin2*: immune response genes; *fit2*, *agpat4*, *dgat2*, *mgll*, *hnf4α*, *scap*, and *cck*: cholesterol and triglycerides metabolism-related gene*.

**Table 3:** Various factors affect gut microbiota of zebrafish.

Factors	Subject	Species/strain	Changes	References
HFD	Zebrafish	*Bacteroidetes*	↑	Arias-Jayo et al., 2018
		*Hyphomicrobium*	↓	
IMZ	Male adult	*Proteobacteria*	↓	Jin et al., 2017
		*Bacteroidetes*	↑	
		*Fusobacteria*	↑	
		*Firmicutes*	↑	
MeHgCl	Adult	*Bacteroidetes*	↓	Zhu et al., 2020
		*Proteobacteria*	↓	
GFD	Zebrafish	*Legionellales*	↑	Koo et al., 2017
		*Rhizobiaceae*	↑	
		*Rhodobacter*	↑	
MC-LR	Zebrafish	*Actinobacteria*	↑	Li et al., 2019
		*Lactobacillus*	↑	
Pb	Adult male	*α-Proteobacteria*	↓	Xia et al., 2018
		*Firmicutes*	↑	
GFMs	Zebrafish	*Fusobacteria*	↑	Zheng et al., 2019
		*Cetobacterium*	↑	
		*Lactobacillus*	↑	
		*Firmicutes*	↓	
		*Pseudomonas*	↓	
*M. aeruginosa*	Zebrafish	*Shewanella*	↑	Qian et al., 2019

*HFD, high-fat diet; IMZ, fungicide imazalil; GFD, gluten formulated diet; MC-LR, microcystin-LR; GFMs, graphene-family materials; and *M. aeruginosa*, *Microcystis aeruginosa**.

## Conclusion and Outlook

Zebrafish has more potential and opportunities to provide a more comprehensive understanding the interactions of gut microbiota and host to help us establish the relationship between gut microbiota and host health (Rawls et al., 2006; Chen et al., 2018; Zheng et al., 2019; Jia et al., 2021). But several factors will limit the use of zebrafish in gut microbiology research.

First, it is believed that the composition of gut microbiota and its metabolites and derivatives all play a role in the metabolic homeostasis of the host (Falcinelli et al., 2015), but various endogenous and exogenous factors affect the composition and abundance of the microbiota and it is quite difficult to dissect well the gut microbial-host interactions (Brugman et al., 2009; Roeselers et al., 2011; Mottaz et al., 2017; Yang et al., 2017). Second, although zebrafish are an excellent model organism that has been used to study blood diseases (Haffter et al., 1996; Langenau et al., 2003; Dooley et al., 2008; van Rooijen et al., 2009; Taylor and Zon, 2011; Santoriello and Zon, 2012), cancer (Amatruda et al., 2002; Ignatius and Langenau, 2009; Mione and Trede, 2010; Ceol et al., 2011; Liu and Leach, 2011), heart diseases (Chen et al., 1996; Stainier et al., 1996; Poss et al., 2002; Poss, 2007; Tang et al., 2019; Mukherjee et al., 2021), muscle disorders (Bassett and Currie, 2003; Follo et al., 2013), kidney diseases (Tobin and Beales, 2008; Diep et al., 2011; Sander and Davidson, 2014; Gehrig et al., 2018; Brilli et al., 2019), central nervous system diseases (Stewart et al., 2015; Heylen et al., 2021), and eye diseases (Ohnesorge et al., 2019; Hong and Luo, 2021), zebrafish does have certain disadvantages in mimicking human diseases. These disadvantages include the inclusion of many gene duplications in the zebrafish genome; the phenotypic characteristics of diseases caused by direct homologous genes may differ in zebrafish and humans (Santoriello and Zon, 2012). And the zebrafish genome contains a significant amount of repetitive content of 52.2% (Howe et al., 2013). Therefore, when studying the relationship and interaction of gut microbiota and host with zebrafish, we also need to consider the above-mentioned issues and drawbacks. This will help us to understand the interactions between the gut and the host health better. Third, for zebrafish husbandry, there are many issues currently faced, such as determining a diet that will survive to reproductive maturity, the ability of zebrafish to reproduce over multiple generations, and optimizing precise equipment. Fourth, using zebrafish for gut or gut microbiota studies faces obvious practical challenges, mainly because the gut of zebrafish is small, making it difficult to collect consecutive samples from the same fish and requiring good operator skills. All the above factors may affect the promotion and further application of zebrafish in gut microbiology research.

Due to the limited space of this review, only the roles of gut microbiota in intestinal epithelial cell differentiation, intestinal epithelial proliferation, nutrient metabolism, and immune response regulation are listed. The functions of gut microbiota that affect host health are not comprehensively summarized in this review. Finally, we insist that more efforts should be devoted to study the specific mechanisms by which gut microbiota affect host health and explore their pathways or some associated genetic changes in the future. It is worthwhile to believe that fully exploiting the potential of zebrafish in terms of gut microbiota will help to provide better and more therapeutic options to unravel the pathogenesis and improve host health.

## Author Contributions

PL, JS, and HZ contributed conceptualization. PL and JZ performed the literature search and data analysis. PL wrote the first draft of the manuscript. All authors commented on previous versions of the manuscript. JZ, XL, LG, and YX contributed to writing review and editing. All authors contributed to the article and approved the submitted version.

## Funding

This research was funded by the National Key R&D Program of China, grant number 2018YFE0205100, the Science and Technology Plan Project of Chengguan district, Lanzhou, grant number 2019RCCX0071, the Lanzhou Talent Innovation and Entrepreneurship Project, grant number 2019-RC-76, the Scientific Technology Research Projects of Gansu Province, grant number 20JR5RA551, and the Key Program of the National Natural Science Foundation of China, grant number U1632270.

## Conflict of Interest

The authors declare that the research was conducted in the absence of any commercial or financial relationships that could be construed as a potential conflict of interest.

## Publisher’s Note

All claims expressed in this article are solely those of the authors and do not necessarily represent those of their affiliated organizations, or those of the publisher, the editors and the reviewers. Any product that may be evaluated in this article, or claim that may be made by its manufacturer, is not guaranteed or endorsed by the publisher.

## Abbreviations

GF: Germ-free
LPS: Lipopolysaccharide
IAP: Intestinal alkaline phosphatase
CV: Conventionally raised
*fxr*: Farnesoid X receptor
*mcm*: Minichromosome maintenance gene
*pcna*: Proliferating cell nuclear antigen
*Dtymk*: Thymidylate kinase
*Orc4l*: Origin-recognition complex subunit 4
*Rrm2*: Ribonucleotide reductase subunit m2
FFAs: Fatty acids
LDs: Lipid droplets
*Apob*: Apolipoprotein B
TLRs: Toll-like receptors
dpf: Days post-fertilization
IMZ: Imazalil
PBDEs: Polybrominated diphenyl ethers
E2: Estradiol
BPA: Bisphenol A

## References

[ref1] Amatruda J. F. Shepard J. L. Stern H. M. Zon L. I. (2002). Zebrafish as a cancer model system. Cancer Cell 1, 229–231. doi: 10.1016/S1535-6108(02)00052-1, PMID: 12086858

[ref2] Angelucci F. Cechova K. Amlerova J. Hort J. (2019). Antibiotics, gut microbiota, and Alzheimer’s disease. J. Neuroinflammation 16:108. doi: 10.1186/s12974-019-1494-4, PMID: 31118068 PMC6530014

[ref3] Arias-Jayo N. Abecia L. Alonso-Saez L. Ramirez-Garcia A. Rodriguez A. Pardo M. A. (2018). High-fat diet consumption induces microbiota dysbiosis and intestinal inflammation in zebrafish. Microb. Ecol. 76, 1089–1101. doi: 10.1007/s00248-018-1198-9, PMID: 29736898

[ref4] Backhed F. Ding H. Wang T. Hooper L. V. Koh G. Y. Nagy A.. (2004). The gut microbiota as an environmental factor that regulates fat storage. Proc. Natl. Acad. Sci. U. S. A. 101, 15718–15723. doi: 10.1073/pnas.0407076101, PMID: 15505215 PMC524219

[ref5] Bao Z. Zhao Y. Wu A. Lou Z. Lu H. Yu Q.. (2020). Sub-chronic carbendazim exposure induces hepatic glycolipid metabolism disorder accompanied by gut microbiota dysbiosis in adult zebrafish (Daino rerio). Sci. Total Environ. 739:140081. doi: 10.1016/j.scitotenv.2020.140081 32554111

[ref6] Bassett D. I. Currie P. D. (2003). The zebrafish as a model for muscular dystrophy and congenital myopathy. Hum. Mol. Genet. 12, R265–R270. doi: 10.1093/hmg/ddg279 14504264

[ref7] Bates J. M. Akerlund J. Mittge E. Guillemin K. (2007). Intestinal alkaline phosphatase detoxifies lipopolysaccharide and prevents inflammation in zebrafish in response to the gut microbiota. Cell Host Microbe 2, 371–382. doi: 10.1016/j.chom.2007.10.010, PMID: 18078689 PMC2730374

[ref8] Bates J. M. Mittge E. Kuhlman J. Baden K. N. Cheesman S. E. Guillemin K. (2006). Distinct signals from the microbiota promote different aspects of zebrafish gut differentiation. Dev. Biol. 297, 374–386. doi: 10.1016/j.ydbio.2006.05.006, PMID: 16781702

[ref9] Beutler B. Rietschel E. T. (2003). Innate immune sensing and its roots: the story of endotoxin. Nat. Rev. Immunol. 3, 169–176. doi: 10.1038/nri1004, PMID: 12563300

[ref10] Borrelli L. Aceto S. Agnisola C. De Paolo S. Dipineto L. Stilling R. M.. (2016). Probiotic modulation of the microbiota-gut-brain axis and behaviour in zebrafish. Sci. Rep. 6:30046. doi: 10.1038/srep30046, PMID: 27416816 PMC4945902

[ref11] Brilli S. L. Han H. I. Espiritu E. B. Missinato M. A. Rochon E. R. McDaniels M. D.. (2019). Enhancing regeneration after acute kidney injury by promoting cellular dedifferentiation in zebrafish. Dis. Model. Mech. 12:dmm037390. doi: 10.1242/dmm.037390, PMID: 30890583 PMC6505474

[ref12] Brugman S. Liu K. Y. Lindenbergh-Kortleve D. Samsom J. N. Furuta G. T. Renshaw S. A.. (2009). Oxazolone-induced enterocolitis in zebrafish depends on the composition of the intestinal microbiota. Gastroenterology 137, 1757–1767. doi: 10.1053/j.gastro.2009.07.069, PMID: 19698716

[ref13] Brugman S. Schneeberger K. Witte M. Klein M. R. van den Bogert B. Boekhorst J.. (2014). T lymphocytes control microbial composition by regulating the abundance of *Vibrio* in the zebrafish gut. Gut Microbes 5, 737–747. doi: 10.4161/19490976.2014.972228 25536157 PMC4615293

[ref14] Burns A. R. Miller E. Agarwal M. Rolig A. S. Milligan-Myhre K. Seredick S.. (2017). Interhost dispersal alters microbiome assembly and can overwhelm host innate immunity in an experimental zebrafish model. Proc. Natl. Acad. Sci. U. S. A. 114, 11181–11186. doi: 10.1073/pnas.1702511114, PMID: 28973938 PMC5651736

[ref15] Catron T. R. Swank A. Wehmas L. C. Phelps D. Keely S. P. Brinkman N. E.. (2019). Microbiota alter metabolism and mediate neurodevelopmental toxicity of 17beta-estradiol. Sci. Rep. 9:7064. doi: 10.1038/s41598-019-43346-9, PMID: 31068624 PMC6506524

[ref16] Ceol C. J. Houvras Y. Jane-Valbuena J. Bilodeau S. Orlando D. A. Battisti V.. (2011). The histone methyltransferase SETDB1 is recurrently amplified in melanoma and accelerates its onset. Nature 471, 513–517. doi: 10.1038/nature09806, PMID: 21430779 PMC3348545

[ref17] Chen J. N. Haffter P. Odenthal J. Vogelsang E. Brand M. van Eeden F. J.. (1996). Mutations affecting the cardiovascular system and other internal organs in zebrafish. Development 123, 293–302. doi: 10.1242/dev.123.1.293, PMID: 9007249

[ref18] Chen L. Hu C. Lok-Shun L. N. Zhang W. Hua J. Lam P.. (2018). Acute exposure to PBDEs at an environmentally realistic concentration causes abrupt changes in the gut microbiota and host health of zebrafish. Environ. Pollut. 240, 17–26. doi: 10.1016/j.envpol.2018.04.062, PMID: 29729565

[ref19] Chen L. Lam J. Tang L. Hu C. Liu M. Lam P.. (2020). Probiotic modulation of lipid metabolism disorders caused by perfluorobutanesulfonate pollution in zebrafish. Environ. Sci. Technol. 54, 7494–7503. doi: 10.1021/acs.est.0c02345, PMID: 32459962

[ref20] Chiang J. Y. Pathak P. Liu H. Donepudi A. Ferrell J. Boehme S. (2017). Intestinal farnesoid X receptor and takeda G protein couple receptor 5 signaling in metabolic regulation. Dig. Dis. 35, 241–245. doi: 10.1159/000450981, PMID: 28249273 PMC5470086

[ref21] Chu H. Mazmanian S. K. (2013). Innate immune recognition of the microbiota promotes host-microbial symbiosis. Nat. Immunol. 14, 668–675. doi: 10.1038/ni.2635, PMID: 23778794 PMC4109969

[ref22] Claus S. P. Guillou H. Ellero-Simatos S. (2016). The gut microbiota: a major player in the toxicity of environmental pollutants? NPJ Biofilms Microbiomes 2:16003. doi: 10.1038/npjbiofilms.2016.3, PMID: 28721242 PMC5515271

[ref23] Consuegra J. Grenier T. Akherraz H. Rahioui I. Gervais H. Da S. P.. (2020). Metabolic cooperation among commensal bacteria supports drosophila juvenile growth under nutritional stress. iScience 23:101232. doi: 10.1016/j.isci.2020.101232, PMID: 32563155 PMC7305377

[ref24] Coronado M. Solis C. J. Hernandez P. P. Feijoo C. G. (2019). Soybean meal-induced intestinal inflammation in zebrafish is T cell-dependent and has a Th17 cytokine profile. Front. Immunol. 10:610. doi: 10.3389/fimmu.2019.00610, PMID: 31001250 PMC6454071

[ref25] Darby T. M. Naudin C. R. Luo L. Jones R. M. (2020). *Lactobacillus rhamnosus* GG-induced expression of leptin in the intestine orchestrates epithelial cell proliferation. Cell. Mol. Gastroenterol. Hepatol. 9, 627–639. doi: 10.1016/j.jcmgh.2019.12.004, PMID: 31874255 PMC7160578

[ref26] Davenport E. R. Cusanovich D. A. Michelini K. Barreiro L. B. Ober C. Gilad Y. (2015). Genome-wide association studies of the human gut microbiota. PLoS One 10:e140301. doi: 10.1371/journal.pone.0140301, PMID: 26528553 PMC4631601

[ref27] Denison M. S. Whitlock J. J. (1995). Xenobiotic-inducible transcription of cytochrome P450 genes. J. Biol. Chem. 270, 18175–18178. doi: 10.1074/jbc.270.31.18175, PMID: 7629130

[ref28] Diep C. Q. Ma D. Deo R. C. Holm T. M. Naylor R. W. Arora N.. (2011). Identification of adult nephron progenitors capable of kidney regeneration in zebrafish. Nature 470, 95–100. doi: 10.1038/nature09669, PMID: 21270795 PMC3170921

[ref29] Dimitriu P. A. Boyce G. Samarakoon A. Hartmann M. Johnson P. Mohn W. W. (2013). Temporal stability of the mouse gut microbiota in relation to innate and adaptive immunity. Environ. Microbiol. Rep. 5, 200–210. doi: 10.1111/j.1758-2229.2012.00393.x, PMID: 23584963

[ref30] Ding W. Shangguan Y. Zhu Y. Sultan Y. Feng Y. Zhang B.. (2021). Negative impacts of microcystin-LR and glyphosate on zebrafish intestine: linked with gut microbiota and microRNAs? Environ. Pollut. 286:117685. doi: 10.1016/j.envpol.2021.117685, PMID: 34438504

[ref31] Dooley K. A. Fraenkel P. G. Langer N. B. Schmid B. Davidson A. J. Weber G.. (2008). Montalcino, A zebrafish model for variegate porphyria. Exp. Hematol. 36, 1132–1142. doi: 10.1016/j.exphem.2008.04.008, PMID: 18550261 PMC2630115

[ref32] Fabbiano S. Suarez-Zamorano N. Chevalier C. Lazarevic V. Kieser S. Rigo D.. (2018). Functional gut microbiota remodeling contributes to the caloric restriction-induced metabolic improvements. Cell Metab. 28:907. doi: 10.1016/j.cmet.2018.08.005, PMID: 30174308 PMC6288182

[ref33] Falcinelli S. Picchietti S. Rodiles A. Cossignani L. Merrifield D. L. Taddei A. R.. (2015). *Lactobacillus rhamnosus* lowers zebrafish lipid content by changing gut microbiota and host transcription of genes involved in lipid metabolism. Sci. Rep. 5:9336. doi: 10.1038/srep09336, PMID: 25822072 PMC4378510

[ref34] Falcinelli S. Rodiles A. Hatef A. Picchietti S. Cossignani L. Merrifield D. L.. (2017). Dietary lipid content reorganizes gut microbiota and probiotic *L. rhamnosus* attenuates obesity and enhances catabolic hormonal milieu in zebrafish. Sci. Rep. 7:5512. doi: 10.1038/s41598-017-05147-w, PMID: 28717234 PMC5514052

[ref35] Follo C. Ozzano M. Montalenti C. Santoro M. M. Isidoro C. (2013). Knockdown of cathepsin D in zebrafish fertilized eggs determines congenital myopathy. Biosci. Rep. 33:e34. doi: 10.1042/BSR20120100, PMID: 23464837 PMC3616520

[ref36] Franks I. (2013). Microbiota: gut microbes might promote intestinal angiogenesis. Nat. Rev. Gastroenterol. Hepatol. 10:3. doi: 10.1038/nrgastro.2012.227, PMID: 23183793

[ref37] Garcia G. R. Noyes P. D. Tanguay R. L. (2016). Advancements in zebrafish applications for 21st century toxicology. Pharmacol. Ther. 161, 11–21. doi: 10.1016/j.pharmthera.2016.03.009, PMID: 27016469 PMC4851906

[ref38] Gaulke C. A. Martins M. L. Watral V. G. Humphreys I. R. Spagnoli S. T. Kent M. L.. (2019). A longitudinal assessment of host-microbe-parasite interactions resolves the zebrafish gut microbiome’s link to *Pseudocapillaria tomentosa* infection and pathology. Microbiome 7:10. doi: 10.1186/s40168-019-0622-9, PMID: 30678738 PMC6346533

[ref39] Gehrig J. Pandey G. Westhoff J. H. (2018). Zebrafish as a model for drug screening in genetic kidney diseases. Front. Pediatr. 6:183. doi: 10.3389/fped.2018.00183, PMID: 30003073 PMC6031734

[ref40] Gioacchini G. Giorgini E. Merrifield D. L. Hardiman G. Borini A. Vaccari L.. (2012). Probiotics can induce follicle maturational competence: the *Danio rerio* case. Biol. Reprod. 86:65. doi: 10.1095/biolreprod.111.094243, PMID: 22088919 PMC3316265

[ref41] Gomaa E. Z. (2020). Human gut microbiota/microbiome in health and diseases: a review. Antonie Van Leeuwenhoek 113, 2019–2040. doi: 10.1007/s10482-020-01474-7, PMID: 33136284

[ref42] Guo X. Li J. Ran C. Wang A. Xie M. Xie Y.. (2019). Dietary nucleotides can directly stimulate the immunity of zebrafish independent of the intestinal microbiota. Fish Shellfish Immunol. 86, 1064–1071. doi: 10.1016/j.fsi.2018.12.058, PMID: 30590163

[ref43] Haffter P. Granato M. Brand M. Mullins M. C. Hammerschmidt M. Kane D. A.. (1996). The identification of genes with unique and essential functions in the development of the zebrafish, *Danio rerio*. Development 123, 1–36. doi: 10.1242/dev.123.1.1, PMID: 9007226

[ref44] Heylen L. Pham D. De Meulemeester A. Samarut E. Skiba A. Copmans D.. (2021). Pericardial injection of kainic acid induces a chronic epileptic state in larval zebrafish. Front. Mol. Neurosci. 14:753936. doi: 10.3389/fnmol.2021.753936, PMID: 34720874 PMC8551382

[ref45] Hill J. H. Franzosa E. A. Huttenhower C. Guillemin K. (2016). A conserved bacterial protein induces pancreatic beta cell expansion during zebrafish development. elife 5:e20145. doi: 10.7554/eLife.20145, PMID: 27960075 PMC5154760

[ref46] Hohn C. Petrie-Hanson L. (2012). Rag1−/− mutant zebrafish demonstrate specific protection following bacterial re-exposure. PLoS One 7:e44451. doi: 10.1371/journal.pone.0044451, PMID: 22970222 PMC3435260

[ref47] Hong Y. Luo Y. (2021). Zebrafish model in ophthalmology to study disease mechanism and drug discovery. Pharmaceuticals 14:716. doi: 10.3390/ph14080716, PMID: 34451814 PMC8400593

[ref48] Howe K. Clark M. D. Torroja C. F. Torrance J. Berthelot C. Muffato M.. (2013). The zebrafish reference genome sequence and its relationship to the human genome. Nature 496, 498–503. doi: 10.1038/nature12111, PMID: 23594743 PMC3703927

[ref49] Hua Q. Adamovsky O. Vespalcova H. Boyda J. Schmidt J. T. Kozuch M.. (2021). Microbiome analysis and predicted relative metabolomic turnover suggest bacterial heme and selenium metabolism are altered in the gastrointestinal system of zebrafish (*Danio rerio*) exposed to the organochlorine dieldrin. Environ. Pollut. 268:115715. doi: 10.1016/j.envpol.2020.115715, PMID: 33069042

[ref50] Ignatius M. S. Langenau D. M. (2009). Zebrafish as a model for cancer self-renewal. Zebrafish 6, 377–387. doi: 10.1089/zeb.2009.0610, PMID: 19954344 PMC2805034

[ref51] Iqbal J. Hussain M. M. (2009). Intestinal lipid absorption. Am. J. Physiol. Endocrinol. Metab. 296, E1183–E1194. doi: 10.1152/ajpendo.90899.2008, PMID: 19158321 PMC2692399

[ref52] Jault C. Pichon L. Chluba J. (2004). Toll-like receptor gene family and TIR-domain adapters in *Danio rerio*. Mol. Immunol. 40, 759–771. doi: 10.1016/j.molimm.2003.10.001, PMID: 14687933

[ref53] Jeong H. G. Kang M. J. Kim H. G. Oh D. G. Kim J. S. Lee S. K.. (2013). Role of intestinal microflora in xenobiotic-induced toxicity. Mol. Nutr. Food Res. 57, 84–99. doi: 10.1002/mnfr.201200461, PMID: 23166009

[ref54] Jia P. P. Junaid M. Wen P. P. Yang Y. F. Li W. G. Yang X. G.. (2021). Role of germ-free animal models in understanding interactions of gut microbiota to host and environmental health: a special reference to zebrafish. Environ. Pollut. 279:116925. doi: 10.1016/j.envpol.2021.116925, PMID: 33744636

[ref55] Jin C. Luo T. Zhu Z. Pan Z. Yang J. Wang W.. (2017). Imazalil exposure induces gut microbiota dysbiosis and hepatic metabolism disorder in zebrafish. Comp. Biochem. Physiol. C Toxicol. Pharmacol. 202, 85–93. doi: 10.1016/j.cbpc.2017.08.007, PMID: 28888875

[ref56] Jin C. Zeng Z. Fu Z. Jin Y. (2016). Oral imazalil exposure induces gut microbiota dysbiosis and colonic inflammation in mice. Chemosphere 160, 349–358. doi: 10.1016/j.chemosphere.2016.06.105, PMID: 27393971

[ref57] Keith J. W. Dong Q. Sorbara M. T. Becattini S. Sia J. K. Gjonbalaj M.. (2020). Impact of antibiotic-resistant bacteria on immune activation and *Clostridioides difficile* infection in the mouse intestine. Infect. Immun. 88:e00362-19. doi: 10.1128/IAI.00362-19, PMID: 31907198 PMC7093144

[ref58] Kok F. O. Shin M. Ni C. W. Gupta A. Grosse A. S. van Impel A.. (2015). Reverse genetic screening reveals poor correlation between morpholino-induced and mutant phenotypes in zebrafish. Dev. Cell 32, 97–108. doi: 10.1016/j.devcel.2014.11.018, PMID: 25533206 PMC4487878

[ref59] Koo H. Hakim J. A. Powell M. L. Kumar R. Eipers P. G. Morrow C. D.. (2017). Metagenomics approach to the study of the gut microbiome structure and function in zebrafish *Danio rerio* fed with gluten formulated diet. J. Microbiol. Methods 135, 69–76. doi: 10.1016/j.mimet.2017.01.016, PMID: 28167213 PMC5909692

[ref60] Langenau D. M. Traver D. Ferrando A. A. Kutok J. L. Aster J. C. Kanki J. P.. (2003). Myc-induced T cell leukemia in transgenic zebrafish. Science 299, 887–890. doi: 10.1126/science.1080280, PMID: 12574629

[ref61] Lankelma J. M. van Vught L. A. Belzer C. Schultz M. J. van der Poll T. de Vos W. M.. (2017). Critically ill patients demonstrate large interpersonal variation in intestinal microbiota dysregulation: a pilot study. Intensive Care Med. 43, 59–68. doi: 10.1007/s00134-016-4613-z, PMID: 27837233 PMC5203863

[ref62] Lefebvre P. Cariou B. Lien F. Kuipers F. Staels B. (2009). Role of bile acids and bile acid receptors in metabolic regulation. Physiol. Rev. 89, 147–191. doi: 10.1152/physrev.00010.2008, PMID: 19126757

[ref63] Ley R. E. Hamady M. Lozupone C. Turnbaugh P. J. Ramey R. R. Bircher J. S.. (2008). Evolution of mammals and their gut microbes. Science 320, 1647–1651. doi: 10.1126/science.1155725, PMID: 18497261 PMC2649005

[ref64] Li J. Chen C. Zhang T. Liu W. Wang L. Chen Y.. (2019). μEvaluation of microcystin-LR absorption using an in vivo intestine model and its effect on zebrafish intestine. Aquat. Toxicol. 206, 186–194. doi: 10.1016/j.aquatox.2018.11.014, PMID: 30496952

[ref65] Liu S. Leach S. D. (2011). Zebrafish models for cancer. Annu. Rev. Pathol. 6, 71–93. doi: 10.1146/annurev-pathol-011110-130330, PMID: 21261518

[ref66] Liu Y. Yao Y. Li H. Qiao F. Wu J. Du Z.. (2016). Influence of endogenous and exogenous estrogenic endocrine on intestinal microbiota in zebrafish. PLoS One 11:e163895. doi: 10.1371/journal.pone.0168743, PMID: 27701432 PMC5049800

[ref67] Lopez N. A. Ikeda-Ohtsubo W. Sipkema D. Peggs D. McGurk C. Forlenza M.. (2020). Feed, microbiota, and gut immunity: using the zebrafish model to understand fish health. Front. Immunol. 11:114. doi: 10.3389/fimmu.2020.00039, PMID: 32117265 PMC7014991

[ref68] Lozupone C. A. Stombaugh J. I. Gordon J. I. Jansson J. K. Knight R. (2012). Diversity, stability and resilience of the human gut microbiota. Nature 489, 220–230. doi: 10.1038/nature11550, PMID: 22972295 PMC3577372

[ref69] Mangiola F. Ianiro G. Franceschi F. Fagiuoli S. Gasbarrini G. Gasbarrini A. (2016). Gut microbiota in autism and mood disorders. World J. Gastroenterol. 22, 361–368. doi: 10.3748/wjg.v22.i1.361, PMID: 26755882 PMC4698498

[ref70] Milligan-Myhre K. Charette J. R. Phennicie R. T. Stephens W. Z. Rawls J. F. Guillemin K.. (2011). Study of host-microbe interactions in zebrafish. Methods Cell Biol. 105, 87–116. doi: 10.1016/B978-0-12-381320-6.00004-7 21951527 PMC4700925

[ref71] Mione M. C. Trede N. S. (2010). The zebrafish as a model for cancer. Dis. Model. Mech. 3, 517–523. doi: 10.1242/dmm.004747, PMID: 20354112 PMC2931530

[ref72] Mottaz H. Schonenberger R. Fischer S. Eggen R. Schirmer K. Groh K. J. (2017). Dose-dependent effects of morphine on lipopolysaccharide (LPS)-induced inflammation, and involvement of multixenobiotic resistance (MXR) transporters in LPS efflux in teleost fish. Environ. Pollut. 221, 105–115. doi: 10.1016/j.envpol.2016.11.046, PMID: 28010888

[ref73] Muegge B. D. Kuczynski J. Knights D. Clemente J. C. Gonzalez A. Fontana L.. (2011). Diet drives convergence in gut microbiome functions across mammalian phylogeny and within humans. Science 332, 970–974. doi: 10.1126/science.1198719, PMID: 21596990 PMC3303602

[ref74] Mukherjee D. Wagh G. Mokalled M. H. Kontarakis Z. Dickson A. L. Rayrikar A.. (2021). Ccn2a is an injury-induced matricellular factor that promotes cardiac regeneration in zebrafish. Development 148:dev193219. doi: 10.1242/dev.193219, PMID: 33234717 PMC7847265

[ref75] Murdoch C. C. Rawls J. F. (2019). Commensal microbiota regulate vertebrate innate immunity-insights from the zebrafish. Front. Immunol. 10:2100. doi: 10.3389/fimmu.2019.02100, PMID: 31555292 PMC6742977

[ref76] Ohnesorge N. Sasore T. Hillary D. Alvarez Y. Carey M. Kennedy B. N. (2019). Orthogonal drug pooling enhances phenotype-based discovery of ocular antiangiogenic drugs in zebrafish larvae. Front. Pharmacol. 10:508. doi: 10.3389/fphar.2019.00508, PMID: 31178719 PMC6544088

[ref77] Osimani A. Milanovic V. Roncolini A. Riolo P. Ruschioni S. Isidoro N.. (2019). *Hermetia illucens* in diets for zebrafish (*Danio rerio*): a study of bacterial diversity by using PCR-DGGE and metagenomic sequencing. PLoS One 14:e225956. doi: 10.1371/journal.pone.0225956, PMID: 31821372 PMC6903733

[ref78] Patterson E. Ryan P. M. Cryan J. F. Dinan T. G. Ross R. P. Fitzgerald G. F.. (2016). Gut microbiota, obesity and diabetes. Postgrad. Med. J. 92, 286–300. doi: 10.1136/postgradmedj-2015-133285, PMID: 26912499

[ref79] Poss K. D. (2007). Getting to the heart of regeneration in zebrafish. Semin. Cell Dev. Biol. 18, 36–45. doi: 10.1016/j.semcdb.2006.11.009, PMID: 17178459

[ref80] Poss K. D. Wilson L. G. Keating M. T. (2002). Heart regeneration in zebrafish. Science 298, 2188–2190. doi: 10.1126/science.1077857, PMID: 12481136

[ref81] Qian H. Zhang M. Liu G. Lu T. Sun L. Pan X. (2019). Effects of different concentrations of *Microcystis aeruginosa* on the intestinal microbiota and immunity of zebrafish (*Danio rerio*). Chemosphere 214, 579–586. doi: 10.1016/j.chemosphere.2018.09.156, PMID: 30286424

[ref82] Ran C. Hu J. Liu W. Liu Z. He S. Bui C. T. D.. (2016). Thymol and carvacrol affect hybrid tilapia through the combination of direct stimulation and an intestinal microbiota-mediated effect: insights from a germ-free zebrafish model. J. Nutr. 146, 1132–1140. doi: 10.3945/jn.115.229377, PMID: 27075912

[ref83] Rawls J. F. Mahowald M. A. Ley R. E. Gordon J. I. (2006). Reciprocal gut microbiota transplants from zebrafish and mice to germ-free recipients reveal host habitat selection. Cell 127, 423–433. doi: 10.1016/j.cell.2006.08.043, PMID: 17055441 PMC4839475

[ref84] Rawls J. F. Samuel B. S. Gordon J. I. (2004). Gnotobiotic zebrafish reveal evolutionarily conserved responses to the gut microbiota. Proc. Natl. Acad. Sci. U. S. A. 101, 4596–4601. doi: 10.1073/pnas.0400706101, PMID: 15070763 PMC384792

[ref85] Roeselers G. Mittge E. K. Stephens W. Z. Parichy D. M. Cavanaugh C. M. Guillemin K.. (2011). Evidence for a core gut microbiota in the zebrafish. ISME J. 5, 1595–1608. doi: 10.1038/ismej.2011.38, PMID: 21472014 PMC3176511

[ref86] Sander V. Davidson A. J. (2014). Kidney injury and regeneration in zebrafish. Semin. Nephrol. 34, 437–444. doi: 10.1016/j.semnephrol.2014.06.010, PMID: 25217272

[ref87] Santoriello C. Zon L. I. (2012). Hooked! Modeling human disease in zebrafish. J. Clin. Invest. 122, 2337–2343. doi: 10.1172/JCI60434, PMID: 22751109 PMC3386812

[ref88] Schwarzer M. (2018). Gut microbiota: puppeteer of the host juvenile growth. Curr. Opin. Clin. Nutr. Metab. Care 21, 179–183. doi: 10.1097/MCO.0000000000000463, PMID: 29432296

[ref89] Schwarzer M. Strigini M. Leulier F. (2018). Gut microbiota and host juvenile growth. Calcif. Tissue Int. 102, 387–405. doi: 10.1007/s00223-017-0368-y, PMID: 29214457

[ref90] Semova I. Carten J. D. Stombaugh J. Mackey L. C. Knight R. Farber S. A.. (2012). Microbiota regulate intestinal absorption and metabolism of fatty acids in the zebrafish. Cell Host Microbe 12, 277–288. doi: 10.1016/j.chom.2012.08.003, PMID: 22980325 PMC3517662

[ref91] Sireswar S. Dey G. (2019). Matrix-wise evaluation of in vivo and in vitro efficiencies of *L. rhamnosus* GG-fortified beverages. Food Res. Int. 119, 908–919. doi: 10.1016/j.foodres.2018.10.077, PMID: 30884731

[ref92] Sorbara M. T. Dubin K. Littmann E. R. Moody T. U. Fontana E. Seok R.. (2019). Inhibiting antibiotic-resistant *Enterobacteriaceae* by microbiota-mediated intracellular acidification. J. Exp. Med. 216, 84–98. doi: 10.1084/jem.20181639, PMID: 30563917 PMC6314524

[ref93] Sousa T. Paterson R. Moore V. Carlsson A. Abrahamsson B. Basit A. W. (2008). The gastrointestinal microbiota as a site for the biotransformation of drugs. Int. J. Pharm. 363, 1–25. doi: 10.1016/j.ijpharm.2008.07.009, PMID: 18682282

[ref94] Stagaman K. Burns A. R. Guillemin K. Bohannan B. J. (2017). The role of adaptive immunity as an ecological filter on the gut microbiota in zebrafish. ISME J. 11, 1630–1639. doi: 10.1038/ismej.2017.28, PMID: 28304369 PMC5520148

[ref95] Stainier D. Y. Fouquet B. Chen J. N. Warren K. S. Weinstein B. M. Meiler S. E.. (1996). Mutations affecting the formation and function of the cardiovascular system in the zebrafish embryo. Development 123, 285–292. doi: 10.1242/dev.123.1.285, PMID: 9007248

[ref96] Stappenbeck T. S. Hooper L. V. Gordon J. I. (2002). Developmental regulation of intestinal angiogenesis by indigenous microbes via Paneth cells. Proc. Natl. Acad. Sci. U. S. A. 99, 15451–15455. doi: 10.1073/pnas.202604299, PMID: 12432102 PMC137737

[ref97] Stephens W. Z. Burns A. R. Stagaman K. Wong S. Rawls J. F. Guillemin K.. (2016). The composition of the zebrafish intestinal microbial community varies across development. ISME J. 10, 644–654. doi: 10.1038/ismej.2015.140, PMID: 26339860 PMC4817687

[ref98] Stephens W. Z. Wiles T. J. Martinez E. S. Jemielita M. Burns A. R. Parthasarathy R.. (2015). Identification of population bottlenecks and colonization factors during assembly of bacterial communities within the zebrafish intestine. MBio 6, e1115–e1163. doi: 10.1128/mBio.01163-15 PMC4626852 26507229

[ref99] Stewart A. M. Gerlai R. Kalueff A. V. (2015). Developing highER-throughput zebrafish screens for in-vivo CNS drug discovery. Front. Behav. Neurosci. 9:14. doi: 10.3389/fnbeh.2015.00014, PMID: 25729356 PMC4325915

[ref100] Stressmann F. A. Bernal-Bayard J. Perez-Pascual D. Audrain B. Rendueles O. Briolat V.. (2021). Mining zebrafish microbiota reveals key community-level resistance against fish pathogen infection. ISME J. 15, 702–719. doi: 10.1038/s41396-020-00807-8, PMID: 33077888 PMC8027185

[ref101] Sun Y. Tang L. Liu Y. Hu C. Zhou B. Lam P.. (2019). Activation of aryl hydrocarbon receptor by dioxin directly shifts gut microbiota in zebrafish. Environ. Pollut. 255:113357. doi: 10.1016/j.envpol.2019.113357, PMID: 31671369

[ref102] Swain P. Nayak S. K. Nanda P. K. Dash S. (2008). Biological effects of bacterial lipopolysaccharide (endotoxin) in fish: a review. Fish Shellfish Immunol. 25, 191–201. doi: 10.1016/j.fsi.2008.04.009, PMID: 18603445

[ref103] Tang W. Martik M. L. Li Y. Bronner M. E. (2019). Cardiac neural crest contributes to cardiomyocytes in amniotes and heart regeneration in zebrafish. elife 8:47929. doi: 10.7554/eLife.47929, PMID: 31393264 PMC6721792

[ref104] Taylor A. M. Zon L. I. (2011). Modeling diamond blackfan anemia in the zebrafish. Semin. Hematol. 48, 81–88. doi: 10.1053/j.seminhematol.2011.02.002, PMID: 21435504

[ref105] Tian Y. Xu J. Feng S. He S. Zhao S. Zhu L.. (2017). The first wave of T lymphopoiesis in zebrafish arises from aorta endothelium independent of hematopoietic stem cells. J. Exp. Med. 214, 3347–3360. doi: 10.1084/jem.20170488, PMID: 28931624 PMC5679161

[ref106] Tobin J. L. Beales P. L. (2008). Restoration of renal function in zebrafish models of ciliopathies. Pediatr. Nephrol. 23, 2095–2099. doi: 10.1007/s00467-008-0898-7, PMID: 18604564 PMC7462901

[ref107] Ubeda C. Taur Y. Jenq R. R. Equinda M. J. Son T. Samstein M.. (2010). Vancomycin-resistant *Enterococcus* domination of intestinal microbiota is enabled by antibiotic treatment in mice and precedes bloodstream invasion in humans. J. Clin. Invest. 120, 4332–4341. doi: 10.1172/JCI43918, PMID: 21099116 PMC2993598

[ref108] Udayangani R. Dananjaya S. Nikapitiya C. Heo G. J. Lee J. De Zoysa M. (2017). Metagenomics analysis of gut microbiota and immune modulation in zebrafish (*Danio rerio*) fed chitosan silver nanocomposites. Fish Shellfish Immunol. 66, 173–184. doi: 10.1016/j.fsi.2017.05.018, PMID: 28479399

[ref109] van Rooijen E. Voest E. E. Logister I. Korving J. Schwerte T. Schulte-Merker S.. (2009). Zebrafish mutants in the von Hippel-Lindau tumor suppressor display a hypoxic response and recapitulate key aspects of Chuvash polycythemia. Blood 113, 6449–6460. doi: 10.1182/blood-2008-07-167890, PMID: 19304954

[ref110] Vitek L. Haluzik M. (2016). The role of bile acids in metabolic regulation. J. Endocrinol. 228, R85–R96. doi: 10.1530/JOE-15-0469, PMID: 26733603

[ref111] Wang B. Zhang S. Q. Dong J. L. Li Y. Jin Y. X. Xiao H. W.. (2022). Ambient temperature structures the gut microbiota of zebrafish to impact the response to radioactive pollution. Environ. Pollut. 293:118539. doi: 10.1016/j.envpol.2021.118539, PMID: 34798219

[ref112] Wen J. Mercado G. P. Volland A. Doden H. L. Lickwar C. R. Crooks T.. (2021). Fxr signaling and microbial metabolism of bile salts in the zebrafish intestine. Sci. Adv. 7:eabg1371. doi: 10.1126/sciadv.abg1371, PMID: 34301599 PMC8302129

[ref113] Willing B. P. Russell S. L. Finlay B. B. (2011). Shifting the balance: antibiotic effects on host-microbiota mutualism. Nat. Rev. Microbiol. 9, 233–243. doi: 10.1038/nrmicro2536, PMID: 21358670

[ref114] Wong S. Stephens W. Z. Burns A. R. Stagaman K. David L. A. Bohannan B. J.. (2015). Ontogenetic differences in dietary fat influence microbiota assembly in the zebrafish gut. MBio 6, e615–e687. doi: 10.1128/mBio.00687-15 PMC4611033 26419876

[ref115] Xia J. Lu L. Jin C. Wang S. Zhou J. Ni Y.. (2018). Effects of short term lead exposure on gut microbiota and hepatic metabolism in adult zebrafish. Comp. Biochem. Physiol. C Toxicol. Pharmacol. 209, 1–8. doi: 10.1016/j.cbpc.2018.03.007, PMID: 29574035

[ref116] Xin G. Y. Li W. G. Suman T. Y. Jia P. P. Ma Y. B. Pei D. S. (2020). Gut bacteria *Vibrio* sp. and *Aeromonas* sp. trigger the expression levels of proinflammatory cytokine: first evidence from the germ-free zebrafish. Fish Shellfish Immunol. 106, 518–525. doi: 10.1016/j.fsi.2020.08.018, PMID: 32810528

[ref117] Yang H. T. Zou S. S. Zhai L. J. Wang Y. Zhang F. M. An L. G.. (2017). Pathogen invasion changes the intestinal microbiota composition and induces innate immune responses in the zebrafish intestine. Fish Shellfish Immunol. 71, 35–42. doi: 10.1016/j.fsi.2017.09.075, PMID: 28964859

[ref118] Yoo J. Y. Groer M. Dutra S. Sarkar A. McSkimming D. I. (2020). Gut microbiota and immune system interactions. Microorganisms 8:1587. doi: 10.3390/microorganisms8101587, PMID: 33076307 PMC7602490

[ref119] Zang L. Ma Y. Huang W. Ling Y. Sun L. Wang X.. (2019). Dietary *Lactobacillus plantarum* ST-III alleviates the toxic effects of triclosan on zebrafish (*Danio rerio*) via gut microbiota modulation. Fish Shellfish Immunol. 84, 1157–1169. doi: 10.1016/j.fsi.2018.11.007, PMID: 30423455

[ref120] Zhang Q. L. Li H. W. Wu W. Zhang M. Guo J. Deng X. Y.. (2019). The response of microbiota community to *Streptococcus agalactiae* infection in zebrafish intestine. Front. Microbiol. 10:2848. doi: 10.3389/fmicb.2019.02848, PMID: 31866993 PMC6908962

[ref121] Zhang J. Meng H. Kong X. Cheng X. Ma T. He H.. (2021). Combined effects of polyethylene and organic contaminant on zebrafish (*Danio rerio*): accumulation of 9-Nitroanthracene, biomarkers and intestinal microbiota. Environ. Pollut. 277:116767. doi: 10.1016/j.envpol.2021.116767, PMID: 33640823

[ref122] Zhang Z. Ran C. Ding Q. W. Liu H. L. Xie M. X. Yang Y. L.. (2019). Ability of prebiotic polysaccharides to activate a HIF1alpha-antimicrobial peptide axis determines liver injury risk in zebrafish. Commun. Biol. 2:274. doi: 10.1038/s42003-019-0526-z, PMID: 31372513 PMC6658494

[ref123] Zhang H. Sparks J. B. Karyala S. V. Settlage R. Luo X. M. (2015). Host adaptive immunity alters gut microbiota. ISME J. 9, 770–781. doi: 10.1038/ismej.2014.165, PMID: 25216087 PMC4331585

[ref124] Zheng M. Lu J. Lin G. Su H. Sun J. Luan T. (2019). Dysbiosis of gut microbiota by dietary exposure of three graphene-family materials in zebrafish (*Danio rerio*). Environ. Pollut. 254:112969. doi: 10.1016/j.envpol.2019.112969, PMID: 31398638

[ref125] Zhou L. Limbu S. M. Shen M. Zhai W. Qiao F. He A.. (2018). Environmental concentrations of antibiotics impair zebrafish gut health. Environ. Pollut. 235, 245–254. doi: 10.1016/j.envpol.2017.12.073, PMID: 29291524

[ref126] Zhu J. Tang L. Qiao S. Wang L. Feng Y. Wang L.. (2020). Low-dose methylmercury exposure impairs the locomotor activity of zebrafish: role of intestinal inositol metabolism. Environ. Res. 190:110020. doi: 10.1016/j.envres.2020.110020, PMID: 32777273

